# Editorial on Special Issue “Artificial Intelligence in Pathological Image Analysis”

**DOI:** 10.3390/diagnostics13050828

**Published:** 2023-02-21

**Authors:** Masayuki Tsuneki

**Affiliations:** Medmain Research, Medmain Inc., 2-4-5-104, Akasaka, Chuo-ku, Fukuoka 810-0042, Japan; tsuneki@medmain.com; Tel.: +81-92-707-1977

The artificial intelligence (AI), especially deep learning models, is highly compatible with medical images and natural language processing and is expected to be applied to pathological image analysis and other medical fields. In routine pathological diagnosis, the histopathological and cytopathological examination of specimens is conventionally performed under a light microscope. Whole slide images (WSIs) are the digitized counterparts of the conventional glass slides obtained using specialized scanning devices. In recent years, digital pathology has been steadily introduced into clinical workflows, such as intraoperative consultations and secondary consultations. Pathology diagnosis support systems (computer-aided detection/diagnosis: CAD) using AI are useful for various classification tasks, such as histopathological subtyping, tumor grading, immunohistochemical scoring, and predictions of genetic mutation and protein expression profiles [1]. It is becoming possible to develop AI that can not only perform image classification and detection tasks, but also infer histopathological findings from images by combining pathological images with natural language. In a time of distinct paradigm shifts and novel technological innovations, it is necessary for us to establish a unified comprehension(s) of AI approaches in experimental and clinical pathology. In this Special Issue “Artificial Intelligence in Pathological Image Analysis”, we collected a review and thirteen research articles in the areas of AI models in clinical and experimental pathology and computer vision in pathological image analysis. The published studies in this Special Issue provide great insights into the latest knowledge about the application of AI for pathological image analysis.

Kim et al. summarized the current trends and challenges to the application of AI in pathology [2]. In this review article, the authors described the development of computational pathology (CPATH), its applicability to AI development, and the challenges it faces, such as algorithm validation and interpretability, computing systems, reimbursement, ethics, and regulations. Further, the authors presented an overview of novel AI-based approaches that could be integrated into the pathology laboratory workflow. As the authors described, explainable AI and ethics and security issues are important topics in CPATH. To develop safe and reliable AI, the pathology community needs more clinical research and laboratory practices. This review paper provides the current research status of AI in pathology and future perspectives for successful applications.

Our research article demonstrated a deep learning model for prostate adenocarcinoma classification in core needle biopsy WSIs using transfer learning [3]. In routine clinical practice, diagnosing 12 core biopsy specimens using a microscope is time-consuming, manual process, and it is limited in terms of human resources. The authors trained deep learning models capable of classifying core needle biopsy WSIs into adenocarcinoma and benign (non-neoplastic) lesions and achieved an ROC-AUC of up to 0.978 in the core needle biopsy test sets for adenocarcinoma. Deep learning-based computational algorithms might be useful as routine histopathological diagnostic aids for prostate adenocarcinoma classification in core needle biopsy specimens.

Rakovic et al. conducted a survey of prostate cancer UK supporters for the use of digital pathology and AI in the histopathological diagnosis of prostate cancer [4]. A total of 1276 responses to the online survey were analyzed. It was revealed that most of the respondents were in favor of advances in prostate cancer diagnosis by means of digital pathology and AI-assisted diagnostics as adjuncts to current clinical workflows. However, a small minority of them were not in favor of the use of AI in histopathology for reasons which are not easily addressed. Importantly, the patients are more comfortable with the overall responsibility for a histopathology report remaining with the histopathologist and relying on their decision making to use AI and integrate its findings into the final report.

Baek et al. demonstrated the AI-assisted image analysis of acetaminophen-induced acute hepatic injury in Sprague-Dawley rats [5]. The aim of this study was to apply deep learning models for the assessment of toxicological pathology in a non-clinical study. Authors trained the model for various hepatic lesions, including necrosis, inflammation, infiltration, and portal triad at the WSI level. The deep learning model achieved an overall model accuracy of 96.44%. Importantly, the model predicted lesions of portal triad, necrosis, and inflammation with high correlations with annotated lesions by toxicologic pathologists. This study suggested that the deep learning algorithm (Mask R-CNN algorithm) can be applied to implement diagnosis and prediction of hepatic lesions in toxicological pathology.

Zurac et al. developed the AI-based method for identifying mycobacterium tuberculosis in Ziehl–Neelsen-stained tissue specimen WSIs [6]. In routine histopathological diagnosis, detecting mycobacterium tuberculosis in Ziehl–Neelsen-stained slides is difficult and time consuming because of the bacillus size. The developed deep learning model achieved an ROC-AUC of 0.977, an accuracy of 98.33%, a sensitivity of 95.65%, and a specificity of 100% for identifying mycobacterium tuberculosis bacilli on WSIs, which were better than or similar to those data of a team of pathologists who manually inspected slides and WSIs. By using the developed deep learning algorithm, the pathologists saved at least one-third of the total examining time.

Park et al. proposed a new training method called MixPatch, which was designed to improve a CNN-based classifier by specifically addressing the prediction uncertainty problem and examine its effectiveness at improving the diagnosis performance in the context of histopathological image analysis [7]. MixPatch generates and uses a new sub-training dataset, which consists of mixed patches and their pre-defined ground-truth labels. Importantly, by specifically considering the mixed region variation characteristics of the histopathology images, MixPatch augments the extant mixed image methods for medical image analysis, in which the prediction uncertainty is a crucial issue. MixPatch provides a new way to systematically alleviate the overconfidence problem of CNN-based classifiers and improve their prediction accuracy, contributing toward more calibrated and reliable histopathology image analysis.

Serbanescu et al. demonstrated the morphological difference between nodular (low-risk subtype) and micronodular (high-risk subtype) basal cell carcinomas using a classical morphometric approach (a gray-level co-occurrence matrix and histogram analysis) and a deep learning semantic segmentation approach [8]. The authors identified distinct pathological patterns of the tumor component in random fields of the tumor island that did not contain peripheral palisading. They demonstrated that the most significant difference between the morphology of the two (nodular and micronodular) subtypes was represented by the peritumoral cleft component. Importantly, the deep learning semantic segmentation approach provided new insight into the morphologies of nodular and micronodular subtypes of basal cell carcinoma.

Nofallah et al. demonstrated the potential application of the semantic segmentation of clinically important tissue structures for improving the diagnosis of skin biopsy WSIs [9]. It has been revealed that including a clinically important tissue structure along with WSIs improves the learning of the model, especially in challenging diagnostic classes, such as melanoma in situ and invasive melanoma (T1a). The model showed a 6% improvement in the F-score when whole slide images were used along with epidermal nests and cancerous dermal nest segmentation masks compared to that which was achieved using WSIs alone in training and testing the diagnosis pipeline. Importantly, comparing scores with 187 pathologists’ performance on the same test set showed that the model can outperform or have comparable performance in the cases with the aforementioned diagnostic classes.

Legnar et al. investigated the possibility to predict a final diagnosis based on a written neuropathological description using various natural language processing (NLP) methods [10]. Certain diagnoses or groups of diagnoses (e.g., amyloid-deposition-associated diseases) could be predicted very well; however, in several cases, the morphological description was apparently not sufficient to make accurate predictions. This is because some diagnoses are associated with one pattern, but for others, there is a complex pattern combination which makes the prediction difficult without patho-physiological knowledge. Overall, it has been revealed that the morphological description texts, used as a surrogate for image analysis, enable the correct diagnosis to be achieved for some entities.

Cazzato et al. trained the fast random forest (FRF) algorithm to be able to support the specialist to automatically highlight the anomalous pixel regions and to estimate a possible risk by quantifying the percentage of these regions with atypical morphological features starting from routine histopathological images [11]. An important tool for melanoma diagnosis is the probability image estimated by the processed FRF output image. The probability image is useful to discriminate between information about ambiguous lesions. The FRF algorithm proved to be successful, with a discordance of 17% with respect to the results of the dermatopathologist, meaning that this type of supervised algorithm to can help the dermatopathologist in achieving the challenging diagnosis of malignant melanoma.

VanBerlo et al. developed a deep learning solution for automatic lung ultrasound view annotation that effectively improves the efficiency of downstream annotation tasks, which can distinguish between parenchymal and pleural lung ultrasound views with 92.5% accuracy [12]. The automatic partitioning of a 780 clip lung ultrasound dataset by view led to a 42 min reduction of the downstream manual annotation time and resulted in the production of 55 ± 6 extra relevant labels per hour. This deep learning-based automated tool considerably improved the annotation efficiency, resulting in a higher throughput relevant to the annotating task at hand, which can be applied to other unannotated datasets to save considerable manual annotation time and effort.

Kawazoe et al. demonstrated an automated computational pipeline to detect glomeruli and to segment the histopathological regions inside of the glomerulus in a WSI [13]. The computational pipeline automatically detects glomeruli on PAS-stained WSIs, followed by segmenting the Bowman’s space, the glomerular tuft, the crescentic, and the sclerotic region inside of the glomeruli. To predict the estimated glomerular filtration rate (eGFR) in cases of immunoglobulin A nephropathy (IgAN), it is important to quantify the sclerotic region using the developed pipeline. Importantly, the developed automated computational pipeline could aid in diagnosing renal pathology by visualizing and quantifying the histopathological feature of the glomerulus and potentially accelerate the research in order to better understand the prognosis of IgAN.

Fauzi et al. demonstrated a cell detection and classification system based on a deep learning model for use with the Allred scoring system for breast carcinoma hormone receptor status testing [14]. The computational pipeline first detects all of the cells within the specific regions and classifies them into negatively, weakly, moderately, and strongly stained ones, followed by Allred scoring for the estrogen receptor (ER) status evaluation on WSIs. The automated Allred scores matches well with pathologists’ scores for both the actual Allred score and hormonal treatment cases. The proposed system can automate the exhaustive exercise to provide fast and reliable assistance to pathologists and medical personnel.

Palm et al. examined the performance of a digitalized and artificial intelligence (AI)-assisted workflow for HER2 status determination in accordance with the American Society of Clinical Oncology (ASCO)/College of Pathologists (CAP) guidelines [15]. The HER2 4B5 algorithm in the uPath enterprise software and the HER2 Dual ISH image analysis algorithm (Roche Diagnostic International, Rotkreuz, Switzerland) were used in this study. The authors demonstrated the feasibility of a combined HER2 IHC and ISH AI workflow in the primary and metastatic breast cancers, with a Cohen’s κ of 0.94 when it was assessed in accordance with the ASCO/CAP recommendations.

In summary, in this Special Issue, there are wide varieties of valuable scientific papers including a review article and papers on deep learning models in pathological applications, human and toxicological pathology, and various methodologies. AI-based computational algorithms, including deep learning models, are taking digital pathology beyond mere digitization and telepathology [1]. The incorporation of AI-based computer vision and natural language processing algorithms in routine clinical workflows is on the horizon, reducing processing time and increasing the detection rate of anomalies [1]. It is necessary to continue to share the latest findings and updated methodologies in “Artificial Intelligence in Pathological Image Analysis” and continue to conduct valuable research.
